# A Relação entre a Razão Ácido Úrico/Albumina e a Espessura Média-Intimal da Carótida em Pacientes com Hipertensão

**DOI:** 10.36660/abc.20230691

**Published:** 2024-02-22

**Authors:** Cristian Rodrigues do Nascimento, João Luis Matos Ribeiro, Rodrigo Mendes, Romero Henrique de Almeida Barbosa, Johnnatas Mikael Lopes, Pedro Pereira Tenório

**Affiliations:** 1 Universidade Federal do Vale do Saão Francisco Colegiado de Medicina Paulo Afonso BA Brasil Universidade Federal do Vale do Saão Francisco – Colegiado de Medicina, Paulo Afonso, BA – Brasil; 2 Irmandade Santa Casa de Misericórdia de São Paulo São Paulo SP Brasil Irmandade Santa Casa de Misericórdia de São Paulo, São Paulo, SP – Brasil

**Keywords:** Doenças Cardiovasculares/complicações, Ácido Úrico, Albumina Sérica Humana, Espessura Intima-Média Carotídea, Hipertensão

**Ao Editor**,

Lemos, atentamente, o artigo intitulado: *"A relação entre a razão ácido úrico/albumina e a espessura média-intimal da carótida em pacientes com hipertensão"*, cujo objetivo foi investigar a associação da relação ácido úrico/albumina (RUA) e a espessura média-intimal carotídea (EMIC) em pacientes hipertensos.^1^ Embora o presente estudo tenha demonstrado uma correlação significativa entre RUA e EMIC, com a RUA apresentando maior capacidade discriminativa para valores aumentados de EMIC do que ácido úrico e albumina isolados, e do que os demais marcadores inflamatórios analisados no estudo, como o índice de inflamação imune sistêmica (IIS), a razão neutrófilo/linfócito (RNL), a razão plaqueta/linfócito (RPL) e a razão proteína C reativa/albumina (RCA),^1^ alguns aspectos observados, na leitura do artigo, são dignos de nota.

A classificação dos pacientes em grupos de EMIC aumentada e normal utilizou, como ponto de corte, um valor de 0,9mm, em consonância com o valor de normalidade preconizado nas diretrizes da *European Society of Cardiology* (ESC).^2,3^ Essa simplificação, no entanto, pode levar a erros de classificação em diferentes populações e faixas etárias, nas quais valores semelhantes podem estar dentro da faixa de normalidade.^2^ Isso foi evidenciado por Homma et al.,^4^ mediante o achado de que no envelhecimento o aumento da EMIC pode ser decorrente de um processo de espessamento intimal difuso e não, necessariamente, da formação de placas ateroscleróticas. Diante disso, consideramos importante a estratificação da população estudada por sexo, idade e etnia, tendo em vista a existência de variabilidade entre esses indivíduos, o que pode influenciar uma subestimação dos resultados.

No posicionamento de Ultrassonografia Vascular do Departamento de Imagem Cardiovascular da Sociedade Brasileira de Cardiologia – 2019,^5^ recomenda-se que após aquisição dos dados numéricos da EMI, os valores médios devem ser comparados com valores de referência já existentes, de acordo com as tabelas normativas dos estudos ELSA-Brasil, CAPS ou MESA. A decisão sobre qual tabela utilizar dependerá do gênero, idade e etnia do indivíduo. Deve-se acrescentar na conclusão se a medida se encontra acima ou abaixo do percentil 75, além da tabela utilizada com sua referência bibliográfica. Sendo assim, o ponto de corte utilizado na metodologia pode levar a viés de confusão.

Não obstante, não foi descrito nos grupos a presença de placa carotídea ateromatosa, visto que a identificação da placa é o principal reclassificador de risco cardiovascular. Além do mais, a classificação do estágio da hipertensão no grupo selecionado, conforme a Diretriz de Hipertensão Arterial 2020,^6^ confere um maior risco para lesão de órgão alvo quanto mais elevado os níveis pressóricos, além de maiores disfunção endotelial e remodelamento vascular arterial.

Conforme evidenciado na metodologia, foram excluídos do estudo pacientes com insuficiência cardíaca congestiva, hipertensão secundária, doença cardíaca valvular moderada a grave, doença arterial coronariana, doença renal ou hepática crônica, malignidade, infecção ativa, doença inflamatória crônica, além daqueles que tomavam medicamentos que afetam os níveis séricos de ácido úrico e/ou albumina e aqueles em desnutrição.^1^ No entanto, observamos que indivíduos acometidos por doença arterial obstrutiva periférica não foram inseridos nos critérios de exclusão da pesquisa, o que pode confundir os resultados apresentados, uma vez que as evidências acerca da correlação entre a deterioração aterosclerótica da parede arterial em territórios vasculares periféricos e o aumento da EMIC, já têm sido demonstradas há algumas décadas.^7^ Faz-se necessária, portanto, a consideração dessa comorbidade entre os critérios de exclusão para uma melhor análise dos resultados obtidos no estudo.

Compreendemos, também, necessária a estratificação dos pacientes diabéticos incluídos no estudo em relação à terapêutica antidiabética utilizada, tendo em vista o seu potencial de reduzir a EMIC. Tal efeito foi demonstrado, a título de ilustração, em estudos que analisaram fármacos, como liraglutida^8^ e metformina.^9^ Devido a esses aspectos, estratificar os indivíduos em relação ao hipoglicemiante utilizado, ao tempo de tratamento e à dose empregada, desponta como fator relevante para uma melhor interpretação da associação RUA e EMIC.

Por fim, já se encontra bem estabelecida a relação entre diabetes *mellitus* e calcificação vascular,^10,11^ o que ocorre, entre outros aspectos, pela exposição prolongada das fibras musculares lisas da túnica média à hiperglicemia.^12^ Devido a isso, infere-se que o tempo de doença apresentado pelos indivíduos acometidos por diabetes *mellitus* pode ser um preditor desse processo, além do que a esclerose calcificante da média pode ser mais um confundidor dos resultados apresentados, justificando, pois, a necessidade de estratificar os pacientes diabéticos segundo o tempo de doença.
