# Supplementary material for: A Relação entre a Razão Ácido Úrico/Albumina e a Espessura Média-Intimal da Carótida em Pacientes com Hipertensão
Source: Arq Bras Cardiol. 2024 Feb 22;121(1):e20230691. [Article in Portuguese] doi: 10.36660/abc.20230691 (PMC11081085; doi:10.36660/abc.20230691)
Supplement: Translation

# Translation: The Relationship between Uric Acid/Albumin Ratio and Carotid Intima-Media Thickness in Patients with Hypertension

**Authors:** Cristian Rodrigues do Nascimento, João Luis Matos Ribeiro, Rodrigo Mendes, Romero Henrique de Almeida Barbosa, Johnnatas Mikael Lopes, Pedro Pereira Tenório

**Affiliations:** 1 Paulo Afonso BA Brazil Universidade Federal do Vale do Saão Francisco – Colegiado de Medicina, Paulo Afonso, BA – Brazil; 2 São Paulo SP Brazil Irmandade Santa Casa de Misericórdia de São Paulo, São Paulo, SP – Brazil

**Keywords:** Cardiovascular Diseases/complications, Uric Acid, Human Serum Albumin, Carotid Intima-Media Thickness, Hypertension

**To the Editor**,

We carefully read the article entitled: "*The relationship between the uric acid/albumin ratio and the carotid intima-media thickness in patients with hypertension*", whose objective was to investigate the association of the uric acid/albumin ratio (UAR) and carotid intima-media thickness (CIMT) in hypertensive patients.^1^ Although the present study demonstrated a significant correlation between UAR and CIMT, with UAR showing greater discriminatory capacity for increased CIMT values than uric acid and albumin alone and than the other inflammatory markers analyzed in the study, such as the systemic immune inflammation index (III), the neutrophil/lymphocyte ratio (NLR), the platelet/lymphocyte ratio (PLR) and the C-reactive protein/albumin ratio (CAR),^1^ some aspects observed, when reading the article, are worthy of note.

The classification of patients into groups of increased and normal CIMT used, as a cutoff point, a value of 0.9mm, in line with the normal value recommended in the guidelines of the European Society of Cardiology (ESC).^2,3^ This simplification, however, can lead to classification errors in different populations and age groups, in which similar values may be within the normal range.^2^ This was evidenced by Homma et al.,^4^ through the finding that in aging, the increase in CIMT may be due to a process of diffuse intimal thickening and not necessarily the formation of atherosclerotic plaques. Given this, we consider it important to stratify the studied population by sex, age, and ethnicity, given the existence of variability among these individuals, which may influence an underestimation of the results.

In the Vascular Ultrasonography positioning of the Department of Cardiovascular Imaging of the Brazilian Society of Cardiology – 2019,^5^ it is recommended that after acquiring numerical CIMT data, the average values should be compared with already existing reference values in accordance with the normative tables of the ELSA-Brazil, CAPS or MESA studies. The decision on which table to use will depend on the individual’s gender, age, and ethnicity. It should be added in the conclusion whether the measurement is above or below the 75th percentile, in addition to the table used with its bibliographic reference. Therefore, the cutoff point used in the methodology may lead to confusion bias.

However, the presence of atheromatous carotid plaque was not described in the groups since plaque identification is the main reclassifier of cardiovascular risk. Furthermore, the classification of the stage of hypertension in the selected group, according to the 2020 Arterial Hypertension Guideline,^6^ confers a greater risk for target organ damage the higher the blood pressure levels, in addition to greater endothelial dysfunction and arterial vascular remodeling.

As evidenced in the methodology, patients with congestive heart failure, secondary hypertension, moderate to severe valvular heart disease, coronary artery disease, chronic kidney or liver disease, malignancy, active infection, and chronic inflammatory disease, in addition to those taking medications that affect serum levels of uric acid and/or albumin and those with malnutrition.^1^ However, we observed that individuals affected by peripheral arterial obstructive disease were not included in the research exclusion criteria, which may confuse the results presented since the evidence regarding the correlation between atherosclerotic deterioration of the arterial wall in peripheral vascular territories and the increase in CIMT, have already been demonstrated for some decades.^7^ It is, therefore, necessary to consider this comorbidity among the exclusion criteria for a better analysis of the results obtained in the study.

We also understand that it is necessary to stratify the diabetic patients included in the study in relation to the antidiabetic therapy used, given its potential to reduce CIMT. This effect was demonstrated, by way of illustration, in studies that analyzed drugs such as liraglutide^8^ and metformin.^9^ Due to these aspects, stratifying individuals in relation to the hypoglycemic agent used, the duration of treatment, and the dose used emerges as a relevant factor for a better interpretation of the association between UAR and CIMT.

Finally, the correlation between diabetes mellitus and vascular calcification^10,11^ is well established, which occurs, among other aspects, due to prolonged exposure of the smooth muscle fibers of the tunica media to hyperglycemia.^12^ Due to this, it is inferred that the duration of illness presented by individuals affected by diabetes mellitus can be a predictor of this process, in addition to the fact that calcifying sclerosis of the media can be another confounder of the results presented, thus justifying the need for stratify diabetic patients according to the duration of illness.
