# Supplementary material for: A Relação entre a Razão Ácido Úrico/Albumina e a Espessura Média-Intimal da Carótida em Pacientes com Hipertensão
Source: Arq Bras Cardiol. 2024 Feb 22;121(1):e20230691. [Article in Portuguese] doi: 10.36660/abc.20230691 (PMC11081085; doi:10.36660/abc.20230691)
Supplement: Related texts

# Reply: Carta-resposta

**Authors:** Faysal Şaylık, Tufan Çınar, Murat Selcuk, Ibrahim Halil Tanboğa

**Prezado Editor**,

Agradecemos aos autores por seus valiosos comentários em relação ao nosso estudo intitulado "A relação entre a razão ácido úrico/albumina e a espessura médio-intimal da carótida em pacientes com hipertensão".^1^

Concordamos com os autores na questão de que a idade está correlacionada com a espessura médio-intimal da carótida (EMIC), independentemente da formação de placas ateroscleróticas,^2^ e o grupo de alta EMIC em nosso estudo era mais velho que o grupo de baixa EMIC. No entanto, eliminamos esse fator de confusão adicionando essa variável no modelo multivariável para obter uma razão de chances ajustada para a relação ácido úrico/albumina (RAU) para uma EMIC elevada. Como não houve diferença entre os grupos quanto ao sexo e tal parâmetro também não esteve associado a uma EMIC elevada com base na análise de regressão logística univariada, não inserimos essa variável no modelo multivariável. Finalmente, a população do nosso estudo consistia apenas de etnia caucasiana branca e não havia pacientes de outros grupos étnicos.

O valor de corte para discriminação dos grupos de alta e baixa EMIC foi de 0,9 mm, conforme sugerido pelas diretrizes da Sociedade Europeia de Cardiologia.^3^ Como esse valor de corte também foi utilizado para determinar os pacientes com alto risco cardiovascular na literatura, aplicamos esse valor de corte em nosso estudo.^4^

É uma questão bem conhecida que a presença de placa ateromatosa é um preditor independente de risco cardiovascular.^5^ O estudo teve como objetivo avaliar a relação entre a RAU e a CIMT. Assim, não investigamos a presença de placa de ateroma e seu papel preditivo no risco cardiovascular.

Concordamos com os autores quanto à relação entre o estágio da hipertensão e o risco de danos a órgãos-alvo, incluindo remodelação vascular e disfunção endotelial.^6^ No entanto, a grande maioria dos pacientes já tinha diagnóstico de hipertensão antes da inclusão no estudo e estava em tratamento anti-hipertensivo há algum tempo. Com o auxílio de medicamentos anti-hipertensivos, os valores da pressão arterial foram normalizados na maioria dos pacientes. Com isso, a classificação da hipertensão baseada nas diretrizes recomendadas não foi aplicável em nosso estudo. Portanto, não pudemos avaliar o impacto do grau de hipertensão na EMIC neste estudo. Conforme mencionado pelos autores, há fortes evidências de deterioração arterial periférica e maior EMIC.^7^ Assim, excluímos do estudo os pacientes com doença arterial periférica. Porém, não mencionamos isso no critério de exclusão.

A frequência de pacientes com e sem diabetes mellitus (DM) foi semelhante entre os grupos de alta e baixa EMIC em nosso estudo (p-valor = 0,280). Além disso, o DM não se associou à EMIC elevada na análise de regressão logística univariada. Assim, não houve impacto do DM em ter uma EMIC elevada em nosso estudo. Além disso, pacientes diabéticos com maior duração da doença podem desenvolver complicações micro e macrovasculares. Devido ao difícil cálculo da EMIC nesses pacientes, não incluímos esses pacientes neste estudo. Reconhecemos que os medicamentos antidiabéticos, incluindo a liraglutida e a metformina, podem ter efeitos na EMIC.^8,9^ Por outro lado, inibidores do GLT-1 como a liraglutida têm sido recentemente utilizados para o tratamento do DM em nosso país. Como descrevemos acima, devido à distribuição homogênea dos pacientes diabéticos entre os grupos de estudo, não consideramos que o DM teve um impacto tendencioso na EMIC neste estudo. Entretanto, não coletamos dados referentes à duração do DM, bem como à dosagem e duração dos medicamentos antidiabéticos. Assim, essas limitações podem ser adicionadas na parte de limitações do texto.

## References

[1] Şaylık F, Çınar T, Selçuk M, Tanboğa İH (2023). The Relationship between Uric Acid/Albumin Ratio and Carotid Intima-Media Thickness in Patients with Hypertension. Arq Bras Cardiol.

[2] Homma S, Hirose N, Ishida H, Ishii T, Araki G (2001). Carotid Plaque and Intima-Media Thickness Assessed by B-Mode Ultrasonography in Subjects Ranging from Young Adults to Centenarians. Stroke.

[3] Mancia G, De Backer G, Dominiczak A, Cifkova R, Fagard R, Germano G (2007). 2007 Guidelines for the Management of Arterial Hypertension: The Task Force for the Management of Arterial Hypertension of the European Society of Hypertension (ESH) and of the European Society of Cardiology (ESC). Eur Heart J.

[4] Simon A, Megnien JL, Chironi G (2010). The Value of Carotid Intima-Media Thickness for Predicting Cardiovascular Risk. Arterioscler Thromb Vasc Biol.

[5] Nicholls SJ, Tuzcu EM, Crowe T, Sipahi I, Schoenhagen P, Kapadia S (2006). Relationship between Cardiovascular Risk Factors and Atherosclerotic Disease Burden Measured by Intravascular Ultrasound. J Am Coll Cardiol.

[6] Prakash D (2019). Target Organ Damage in Newly Detected Hypertensive Patients. J Family Med Prim Care.

[7] Allan PL, Mowbray PI, Lee AJ, Fowkes FG (1997). Relationship between Carotid Intima-Media Thickness and Symptomatic and Asymptomatic Peripheral Arterial Disease The Edinburgh Artery Study. Stroke.

[8] Meng XM, Hao YP, Yu S, Ren RZ, Yu X, Tang YX (2023). Effect of Liraglutide on Platelet Distribution Width and Carotid Intima-Media Thickness in Type 2 Diabetic Mellitus Patients with Obesity. Zhonghua Yi Xue Za Zhi.

[9] Chen Y, Li H, Ye Z, Găman MA, Tan SC, Zhu F (2020). The Effect of Metformin on Carotid Intima-Media Thickness (CIMT): A Systematic Review and Meta-Analysis of Randomized Clinical Trials. Eur J Pharmacol.

# Reply

**Authors:** Faysal Şaylık, Tufan Çınar, Murat Selçuk, İbrahim Halil Tanboğa

**Dear Editor**,

We thank to authors for their valuable comments regarding our study entitled "The relationship between the uric acid/albumin ratio and the carotid intima-media thickness in patients with hypertension".^1^

We agree with the authors on the issue that age is correlated with carotid intima-media thickness (CIMT) irrespective of the formation of atherosclerotic plaques,^2^ and the high CIMT group in our study was older than the low CIMT group. However, we eliminated this confounding factor by adding this variable in the multivariable model to get an adjusted odds ratio for uric acid/albumin ratio (UAR) for a high CIMT. Since there was no difference between the groups in terms of gender and such a parameter was also not associated with a high CIMT based on the univariable logistic regression analysis, we did not input that variable in the multivariable model. Finally, the population in our study consisted of only white Caucasian ethnicity, and there were no patients from other ethnic groups.

The cutoff value for the discrimination of high and low CIMT groups was 0.9 mm, as suggested by the guidelines of the European Society of Cardiology.^3^ Because this cutoff value was also used to determine the patients having high cardiovascular risk in the literature, we applied this cutoff value in our study.^4^

It is a well-known issue that the presence of atheromatous plaque is an independent predictor of cardiovascular risk.^5^ The study aimed to evaluate the relationship between the UAR and CIMT. Thus, we did not investigate the presence of atheromatous plaque and its predictive role in cardiovascular risk.

We agree with the authors on the relationship between the stage of hypertension and the risk of target organ damage, including vascular remodeling and endothelial dysfunction.^6^ However, the vast majority of patients had already had hypertension diagnosed before enrollment in the study and were on anti-hypertensive treatment for a while. With the aid of anti-hypertensive drugs, blood pressure values were normalized in most patients. With this, the classification of hypertension based on recommended guidelines was not applicable in our study. Therefore, we could not evaluate the impact of the degree of hypertension on CIMT in this study. As mentioned by the authors, there is strong evidence regarding peripheral arterial deterioration and higher CIMT.^7^ Thus, we excluded the patients with peripheral arterial disease from the study. However, we did not mention it in the exclusion criterion.

The frequency of patients with and without diabetes mellitus (DM) was similar between high and low CIMT groups in our study (p-value = 0.280). Additionally, DM was not associated with high CIMT in the univariable logistic regression analysis. Thus, there was no impact of DM with having a high CIMT in our study. Additionally, diabetic patients with longer disease duration might develop micro- and macro-vascular complications. Due to the difficult calculation of CIMT in such patients, we did not include these patients in this study. We acknowledge that anti-diabetic drugs, including liraglutide and metformin, might have effects on CIMT.^8,9^ On the other hand, GLT-1 inhibitors such as liraglutide have been recently used for the treatment of DM in our country. As we described above, because of the homogeneous distribution of diabetic patients between the study groups, we did not consider that DM had a biased impact on CIMT in this study. However, we did not collect data regarding the duration of DM as well as dosage and the duration of anti-diabetic drugs. Thus, these limitations might be added in the limitations part of the text.
